# 50 Years on, w(h)ither the Ramsar convention? A case of institutional drift

**DOI:** 10.1007/s10531-021-02281-w

**Published:** 2021-08-30

**Authors:** Peter Bridgewater, Rakhyun E. Kim

**Affiliations:** 1 Copernicus Institute of Sustainable Development, Utrecht University, Princetonlaan 8a, 3584 CB Utrecht, The Netherlands; 2 Institute for Applied Ecology and Institute for Governance and Policy Analysis, University of Canberra, 11 Kirinari Street, Bruce, ACT 2617 Australia

**Keywords:** Wetlands, Waterfowl, Institutional drift, Institutional maladaptation, Treaty evolution, Multilateral environmental agreement, Strategic plans

## Abstract

Wetlands have declined in area and quality at an accelerating pace in the last 50 years. Yet, the last 50 years is when international attention has been focussed on wetlands through the Ramsar Convention. An analysis of how the convention has evolved over the past 50 years suggests it has been drifting away from its original mandate in a maladaptive manner, and this drift is a problem for achieving its original objectives. A review of the strategic plans of the convention revealed two key conditions for institutional drifting and the associated lack of success. The first condition lies in its unique situation as a non-UN convention, which reduces the convention’s visibility and interactivity with other biodiversity-related conventions, agencies, or programmes. The second condition is an increasing number of conventions dealing with biodiversity issues, all forcing the Ramsar Convention to seek different roles in an increasingly competitive institutional landscape. A more effective future for the convention arguably lies in reasserting its original mandate, but with cognisance of the changed environmental pressures of the twenty-first century. While this would narrow its increasingly broad focus, such a reorientation will allow wetlands and waterfowl to start a track to recovery, backed by active and focused Contracting Parties in a renewed international convention on wetland conservation, management, and sustainable use.

## Introduction

On 2 February 1971 in Ramsar, Iran, an international meeting agreed text for a convention entitled “Convention on Wetlands of International Importance especially as Waterfowl Habitat” (hereafter Ramsar Convention or the convention). This half-a-century-old Ramsar Convention has often been praised for its near-universal membership (171 Parties), growing list of Wetlands of International Importance, and a successful outreach programme linked to the private sector.

Yet the state of the world’s wetlands has deteriorated in the last 50 years. According to the Global Wetland Outlook (Ramsar Convention on Wetlands 2018), “natural wetlands are in long-term decline around the world; between 1970 and 2015, inland and marine/coastal wetlands both declined by approximately 35%”. It concludes that “wetlands are in serious trouble, declining in area and quality, and under mounting pressure”. This was confirmed in the Global Assessment of the Intergovernmental Platform on Biodiversity and Ecosystem services (IPBES 2019) as “over 85 per cent of wetlands (area) has been lost”.^1^ Why is the convention apparently failing? And are there solutions to this failure? In this paper, we address these questions through an analysis of how the convention has evolved over the past 50 years.

**Fig. 1:**
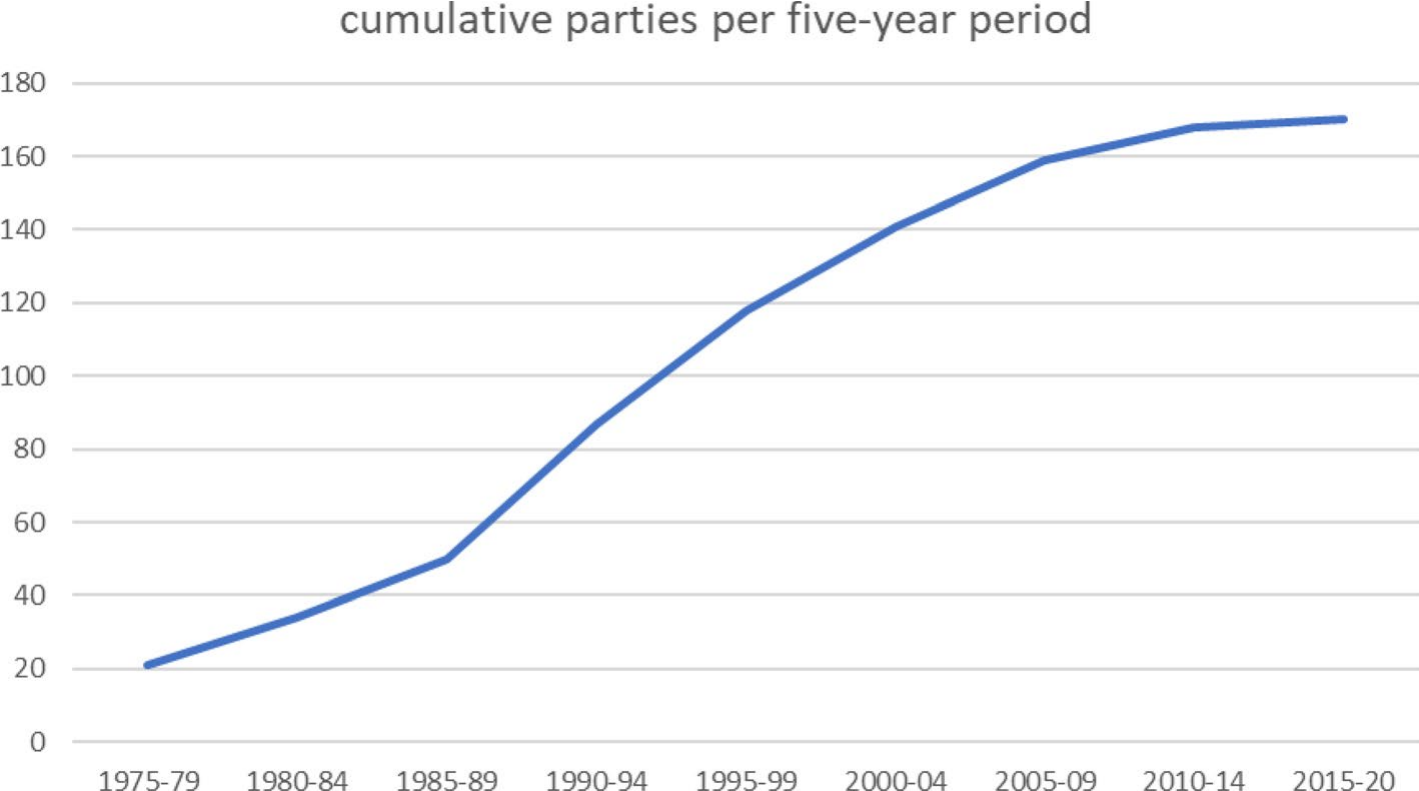
Cumulative adherence of Contracting Parties. The X-axis shows 5-year periods from the convention entering into force, the Y-axis the cumulative number of Parties. This includes succeeding Parties to states such as the USSR and Yugoslavia which have separated into several new nation states, all of which have subsequently acceded in their own right. The data source is the web page of the convention (www.ramsar.org)

The convention has developed in three phases, as shown in Fig. 1. There was an initial pioneer phase from signature in 1971 until the third Conference of the Parties (COP 3^2^) in 1987, with low numbers of Parties adhering and little real action on the ground. This was followed by a building phase of rapid development of activities, greatly increased participation of existing and adherence by new Contracting Parties (until COP X, 2008), and since that time until the present in a consolidation phase, where the remaining nations that are not yet Parties are slowly seeking accession.

Our analytical point of departure is the observation that the visionary aims of the convention’s founders in the 1960s have gradually faded away. The convention’s origin can be traced to the Project MAR (short for “MARshes”, “MARécages”, “MARismas” in English, French and Spanish). Project MAR developed from concern at the rapidly degrading areas of marshland and other wetlands in Europe resulting in a concomitant decline in numbers of migratory waterfowl (Hoffmann 1964). These concerns are crystallised in the convention’s purpose as follows: “to stem the progressive encroachment on and loss of wetlands now and in the future”, particularly “considering the fundamental ecological functions of wetlands as regulators of water regimes and as habitats supporting a characteristic flora and fauna, *especially waterfowl*” (Italic emphasis ours).

Despite those original concerns and the convention title, the current landing page of the convention’s website characterises the convention as “an intergovernmental treaty that provides the framework for national action and international cooperation for the conservation and wise use of wetlands and their resources” (see also Finlayson et al. 2011). Waterfowl have been airbrushed in this description, reflective of moves towards a “water” convention, that began at the 25^th^ Anniversary meeting of COP 6 in 1996. Indeed, the Chair of the Scientific and Technical Review Panel (STRP), at Standing Committee in 2011, noted that the prominence of the theme of water highlights Ramsar’s suitability as “the Water Convention”. While we recognize the need to take cognisance of the changed environmental pressures of the twenty-first century, we observe the convention’s evolution as a case of institutional maladaptation.

Building on Bridgewater and Kim (2021), which reported on some of the key trends in the convention, here, in more detail, we explore the evolution of the Ramsar Convention to understand the underlying dynamics of maladaptation. Based on our participation in, and observation of the convention, we have focused on analysing the convention’s performance through its strategic planning process and resolutions and recommendations of the COP, using available convention documents. Through this analysis of the successive strategic plans developed by the convention, we identify two key conditions for institutional drifting and the associated lack of success: (1) the Ramsar Convention’s unique situation as a non-UN convention, which reduces the convention’s visibility and interactivity with other biodiversity-related conventions, agencies, or programmes; and (2) an increasing number of conventions, agreements and organisations dealing with issues such as migratory species and biodiversity in general, alongside the emergence of the Convention on the Protection and Use of Transboundary Watercourses and International Lakes (UNECE Water Convention) as a global player, all forcing the Ramsar Convention to seek continually changing roles. In other words, we find that the Ramsar Convention has not only *evolved* through deliberate decision-making, but also *drifted* by the collective actions of a larger range of actors than usual, including both external actors and a rich internal *dramatis personae*, interacting with the Parties, and each other. The article concludes with some reflections on how the convention might counter the drift by reasserting its original mandate, providing thus safe spaces for migratory waterfowl, and fulfilling the visionary aims of the convention’s founders in the 1960s.

## The status of the Ramsar convention and its *dramatis personae*

### Building the convention

The MAR conference in 1962 (Hoffmann 1964) focussed on the rapidity with which large stretches of marshland, especially in Europe, were subject to severe modification resulting in decline of migratory waterfowl numbers. It agreed, accordingly, to move for an international convention on the conservation of migratory waterbirds and their supporting wetlands. The draft text proposed at the MAR conference was refined over seven further meetings culminating in the signature process in 1971. In addition to 18 nations, that signature event was also attended by UN specialised agencies (the Food and Agriculture Organization (FAO) and the United Nations Educational, Scientific and Cultural Organization (UNESCO)) and several science-based non-governmental organisations (NGOs).

Under Article 8.1, the convention text allowed that the “International Union for Conservation of Nature and Natural Resources shall perform the continuing bureau duties under this convention”. This was in effect a Secretariat function, although the title was not changed from Bureau to Secretariat until COP IX (2005) (Resolution IX.10). From the outset, the International Waterfowl and Wetlands Research Bureau (IWRB) provided scientific support on waterfowl population data to the International Union for Conservation of Nature (IUCN), and a formal agreement between the IWRB and the IUCN to jointly operate the Bureau was entered into in 1987, persisting until 1993. The IUCN headquarters presently houses the Secretariat and performs basic management functions in support of the Secretariat discharging its duties to the Parties.

The Ramsar Convention, then, from conception to birth in 1971, was a product not simply of intergovernmental action, but of strong partnerships between governments, government agencies, UN specialised agencies, and, especially, international science-based NGOs. Kamigawara (2015) describes this, up until 2008, as conforming to a “managed independence model” of convention development. Yet a more detailed view, especially in the recent decade, suggest the convention is tending more towards a “turbulent non-growth” model, with more and more divergent issues added to the convention’s “to do” list. Table 1 shows this in some detail through the number of resolutions/recommendations of the convention passed at each COP across the three phase of convention evolution.

**Table 1:**
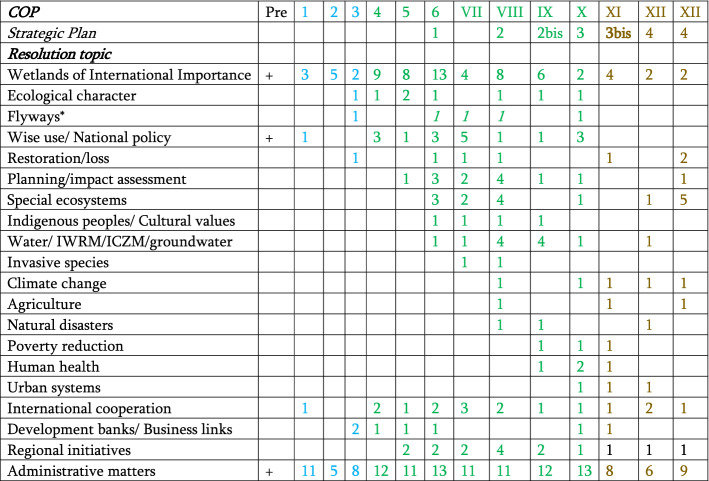
Key topics for which decisions were made across all conferences of the parties

^1^The three phases identified in Fig. 1 are distinguished by colour—*pioneer* in blue*, building* in green and *consolidation* in brown. The establishment period is in black

^2^There were regular resolutions related to CEPA from COP IV, and STRP from COP V, these are subsumed under administrative matters

^3^Several issues were subject to only one resolution: Fisheries (COP IX); Tourism, Biofuels (COPX), Energy (COP XI); Blue carbon, gender (COP XIII)

^4^“Special ecosystems” refers to resolutions dealing with peatlands, coral reefs, karst, rice paddies, intertidal wetlands, and their wise use/conservation

^5^Flyways in general are dealt with only twice by the COP—the italicised resolutions in COP VI-VIII deal specifically with the East Asian–Australasian flyway

^6^Strategic Plans 2 and 3 both had mid-term revisions/restructuring, these revised plans are designated 2 and 3bis

### A UN convention

As the convention predated the existence of the United Nations Environment Programme (UNEP), which currently hosts or coordinates many multilateral environmental agreements, the convention remains, uniquely amongst biodiversity-related conventions, a non-UN treaty. In the twenty-first century, lack of a more formal relationship with the UN system was seen by Parties as increasingly inhibiting effective engagement in global governance processes. The convention’s Standing Committee 26 (2001) discussed developments in international environmental governance, noting important discussions on future arrangements for synergies among the multilateral environmental agreements. The Secretariat had participated in the discussions, but “was displeased by UNEP’s recent proposal for a clustering scheme for biodiversity-related conventions”. This was a missed opportunity, as it would have been the moment for the convention to become part of the UN family of environmental conventions.

Three years on, at COP IX (2005), informal discussions among some Parties floated the idea that UNEP would be a better option than the IUCN to host the Secretariat and give thus full UN status to the convention. While many Parties from developing countries favoured a change to UNEP, several Parties were sceptical about the value of such a move. But this, as noted by Ferrajolo (2011), represents the convention “having reached a certain age, finding application in a legal environment different from the one in which they were conceived”. Standing Committee 35 (2007) agreed to seek the views of the IUCN, UNEP, and UNESCO on the legal status (legal personality) of the Secretariat. In 2008, the Standing Committee 36 again discussed the legal status of the convention and urged continued dialogue with Switzerland, the IUCN, UNEP, and UNESCO with a view to making a recommendation to COP X (2008) on this matter. COP X (2008) was unable to reach consensus and established an open-ended Ad Hoc Working Group to evaluate the success of steps already taken and recommend additional ways of improving the current operations of the convention and its Secretariat to the Standing Committee. On the question of Secretariat hosting, Switzerland favoured the UNEP option and noted that the clustering of biodiversity conventions was under discussion in various fora, and that being administered by UNEP would put the convention on an equal footing with the other biodiversity-related conventions. Despite this view, shared by many Parties, there was sufficient objection from others to prevent consensus. Following further debate, a final decision was taken at the conclusion of COP XI (2012), through Resolution XI.1 “confirming the IUCN as the continuing host for the Secretariat”, while noting the need for “increased involvement in UNEP’s initiatives and programs regarding biodiversity-related conventions to enhance cooperation and synergies between Ramsar and UNEP”.

After such a long and protracted debate, this might be assumed to be the end of the matter. However, the convention is still at a disadvantage with respect to not having observer status at UN bodies, including the UN General Assembly, where environmental issues are increasingly in focus. Led by Uruguay after hosting COP XII (2015), annual attempts have been made to secure such status for the convention, most recently at the 2020 UN General Assembly. But, as with previous efforts, a decision was deferred until the meeting in 2021.

### The dramatis personae

Interactions between NGOs and intergovernmental agencies, as well as the Contracting Parties, have had (Matthews 1993), and continue to have (Bridgewater and Kim 2021), a significant influence on shaping the governance and implementation of the convention. It was the signatory Contracting Parties that became the early key actors on the Ramsar stage, directing much of the early business and thus the structure of the convention, although this took place at a rather leisurely pace. Parties agreed to triennial meetings of a COP and a Standing Committee was also agreed to manage business intersessionally between COPs, through Resolution 3.3. Furthermore, COP 5 (1993) agreed the establishment of a seven-member STRP to give scientific and technical assistance to the Bureau, the Standing Committee and, through them, to the COP.

Amongst the biodiversity-related conventions the Ramsar Convention has the most diverse set of actors that are formally or semi-formally engaged in its work. This echoes back to the nine years of discussion that eventually gave birth to the convention. The major actors in those discussions, with a few exceptions, were not governments but science-focussed international NGOs and intergovernmental organisations. These included the International Council for Bird Preservation (now BirdLife International), the IWRB (now Wetlands International), the IUCN, the WWF, the FAO, and UNESCO. All these organisations, while retaining some science base, are now rather more campaigning and advocacy based, often within the framework of the UN’s 2030 Agenda for Sustainable Development. The Secretariat itself had already foreseen this as way for the convention to develop and expand through resolutions from 2005 dealing with poverty alleviation, human health, and disaster reduction (Tiega 2011).

After 28 years of involvement from genesis, and considerable informal interaction with, and support for, the convention, the COPs gave these International Organization Partners (IOPs) formal recognition through a resolution at COP VII (1998). This resolution (VII.3) “formally confirm[ed] this status for the following organizations: BirdLife International, IUCN-The World Conservation Union, Wetlands International, and the World-Wide Fund for Nature”, subject to a requirement that the relevant organizations entered a formal Memorandum of Cooperation with the convention Secretariat.

The continued importance of IOP interaction in the convention was evident by a Standing Committee 34 (2006) decision that encouraged the Secretariat and IOPs to institutionalize meetings on an annual basis. IOPs were to establish direct contacts with the Parties’ National Focal Points and Parties to include local officers of the IOPs in their National Ramsar/Wetlands Committees. The decision further urged the IOPs to assist Parties, where appropriate, in implementing the Joint Work Plan between the Ramsar Convention and the Convention on Biological Diversity, and it invited the IOPs to report regularly to the Standing Committee on their relevant activities.

Resolution VII.3 also made provision for other suitable organizations to become IOPs, and in 2005 the International Water Management Institute (IWMI) was admitted as an IOP through Resolution IX.16 and in 2015 the Wildfowl and Wetlands Trust (WWT) through Resolution XII.3. IOPs can “participate in an observer capacity and as advisors in all activities of the convention, including the meetings of the Conference of Contracting Parties, the Standing Committee, and the Scientific and Technical Review Panel, as well as regional and subregional meetings”. This offered a degree of participation to these (now six) IOPs unparalleled in other conventions. It did not, of course, exclude regional and national NGOs attending COPs (and Standing Committee) as observers but such organisations must apply for such status at each such meeting, and do not have the same ease of access to convention processes and the Secretariat as the IOPs.

As the number of Parties has grown (Fig. 1), attendance by Parties as observers to Standing Committee has also increased, as has the development of specialist working groups or Advisory Panels. COP IX (2005) agreed establishment of a Management Working Group “to examine and review the various management structures and systems in place within the convention” (Resolution IX.24). Standing Committee already had long-established a sub-group on Finance to review Budget matters. COP IX (2005) also saw the establishment of a Communications, Education, and Public Awareness (CEPA) Oversight Panel (Resolution IX.18). This Panel was to operate cooperatively with the Ramsar Advisory Board on Capacity Building; hosted by the Government of the Netherlands and CEPA activities of Wetlands International. At 35, then, the convention had built several subsidiary bodies to assist its implementation.

## Strategic planning

### The first strategic plan

To explore more fully the evolution of relationships between the key actors in the convention, we use the framework provided by the four strategic plans that have successively been agreed by the COP.

At COP 5 (1993) the Ramsar Bureau was engaged in drafting a “framework for the implementation of the Convention”. The *Kushiro Statement* (Resolution 5.1) incorporated a framework for the implementation of the convention, essentially a stripped-down strategic plan, and a separate framework for Bureau activities. During the next three years discussions continued in the Standing Committee with input from the IWRB (soon to be Wetlands International) on developing an actual strategic plan, which was to have formed part of the 25th Anniversary statement in 1996. But adoption was hardly smooth. Despite intersessional meetings in a Working Group linked to the Subgroup on Finance, the document as presented at COP 6 was far from unanimously agreed. In the run-up to COP 6, the draft was widely discussed at regional meetings and by NGOs. The draft plan was not agreed by the Subgroup on Finance “because of insufficient feedback from the Parties”. These uncertainties, mostly related to budgetary issues, remained in the COP 6 discussions. With considerable reservation, the plan was eventually adopted through Resolution 6.14.

The preambular paragraphs of the plan refer to “serious ongoing and impending threats to many remaining wetlands”—suggesting little had changed for the positive during the convention’s first 25 years. Against that backdrop, the plan, in its introduction, challenged the convention’s Contracting Parties, Standing Committee, STRP, and Bureau to improve the conservation and wise use of wetlands. The plan also emphasised the need for greater involvement of NGOs, beyond the IOPs to assist with implementation at national level. Through the plan it was hoped that technical and policy work of the convention would mesh with the work of the Convention on Biological Diversity and given its involvement with waterfowl, the work of the Convention on Migratory Species. The plan agreed a mission statement for the Convention viz: “the conservation and wise use of wetlands by national action and international cooperation as a means to achieving sustainable development throughout the world”.

### The successive plans

At COP VIII (2002) the second strategic plan for 2003–2008 was agreed in Resolution VIII.25. Running to some 52 pages, this was less a plan than a treatise. It attempted to cram into one document everything the convention was doing, or might do in the future, drawing widely on the outcomes from the World Summit for Sustainable Development (Johannesburg, South Africa, 2002). In familiar wording, the preamble to the Plan noted that despite the many tangible achievements, and even with increasing awareness of the importance of wetland conservation and wise use for human well-being, there was a continuing challenge for the Contracting Parties to ensure conservation and sustainable (wise) use of their wetlands and water resources within the context of global pressures and changes. How had the development of such a prolix strategic plan come about?

The genesis of the plan began at Standing Committee 24 (1999). The plan was to be drafted by 15-member subgroup, including Parties but also IOPs. Drafts were to be prepared for circulation and comments to all Parties, with the third draft more widely circulated to other conventions, relevant intergovernmental institutions, and NGOs active or interested in the convention’s work. The Standing Committee agreed to consult with the Parties in their regions both before and after Standing Committee meetings so that they can fully represent their regions. The degree to which that was effective was somewhat variable. The Standing Committee also urged the IOPs to emulate Wetlands International in devising a complementary work plan based upon that of the Ramsar Bureau.

There was argument concerning the use of quantitative indicators during development of the plan. IOPs, especially the WWF, strongly argued for a site target. The plan finally approved carried the following: “Designation of a further 55 million ha and 250 Ramsar sites, as progress towards global targets of 2500 sites and 250 million ha by 2010”. This was a means of creating leverage on Parties, especially from developing countries, to establish Ramsar sites even where resources for subsequent site management were likely unavailable. Therefore, attempts to promote this target were largely ineffective (Bridgewater 2011). Tickner et al. (2020) note that “formal protection has been inconsistently effective, and there is scope for wider application of lessons from successful protection efforts, such as the involvement of local communities in protected area management”, an issue we discuss later.

Wetlands International, besides hosting a database of Ramsar Sites (the Ramsar Sites Information Service) on behalf of the convention, also proposed “establishment of a Ramsar Wetlands Training and Advisory Service”. The contributions of IOPs were therefore considerable and can be seen effectively to be leading the Parties, and responsible for both the prolix strategic plan and the increase in number of decisions and time spent at COP VIII (2002). Although the plan was adopted through Resolution VIII.25, it needed a second resolution (VIII.26) to identify and establish key implementation targets. The preamble of Resolution VIII.26 discusses a problem—that some developed and, especially, developing countries indicated available resources were not adequate to implement each of the operational objectives of the strategic plan, including those identified as of high priority. Resolution VIII.26 directed the Bureau to prepare a work plan for the convention to give effect to the strategic plan. This showed the difficulties inherent in a strategic plan for the convention, i.e., that much of the implementation lies with Contracting Parties, with some support from actions of IOPs and other wetland-related NGOs, and very little with the Secretariat.

As Parties grappled with the plan’s implementation, its complexity soon became clear, and so by COP IX (2005) a new “framework” to help implement the plan for the second triennium (2006–2008) was agreed. This framework modernised the approach of the plan and reduced its complexity. Besides the three goals that dealt with conservation and wise use of wetlands and water resources (wise use, Ramsar sites, and international cooperation), two were identified as managing the convention (implementation capacity and membership). This clarified considerably the responsibilities of the different elements of the convention—Parties, Secretariat, subsidiary bodies (STRP; CEPA Advisory Panel; Management Review Panel) and the IOPs. It also introduced key result areas (indicators of the effectiveness of the convention’s implementation) and suggested key performance measures to understand the level of achievement, moving the plan to one capable of effectively measuring the convention’s performance.

Standing Committee 34 (2006) established a subgroup to draft the third strategic plan (2009–2015) and oversee the development of a new National Report format for reporting to COP X. Apart from regional representatives from the Parties, BirdLife International was included for the IOPs. This formalised the involvement of the IOPs in the drafting process but also made for a tighter editorial team. The other important aspect was linking the national report format with the strategic plan, bringing planning and reporting tools together. Standing Committee 41 determined that assessments of the strategic plan will be included in the national report format, rather than by using a parallel process of mid-term review.

At COP X (2008) the third strategic plan, set out in Resolution X.1, had five “Goals” like the two previous plans: wise use of wetlands, development of the Ramsar List, international cooperation, implementation capacity, and membership in the convention. In this third plan, however, each goal had a desired and defined outcome and the plan included 28 “strategies” to help reach those outcomes. In part, this was due to Parties increasingly understanding that implementation of the convention was possible only through national actions. This plan had more effective input from Parties in its development than the first two plans and reinforced that IOPs could be more active in working with government officials at national, state, and local levels. The plan further noted the desirability for national environmental governance to shift from sectoral, demand-driven, approaches to an ecosystem approach to policy and decision-making for wetlands and water.

The mission adopted for this third strategic plan—“conservation and wise use of all wetlands through local and national actions and international cooperation, as a contribution towards achieving sustainable development throughout the world”—was essentially the same as that adopted for the first plan in 1997, with two small but important changes: insertion of the word “local” before national implies the need for the convention to be enacted at wide scale, and puts onus on Parties to ensure this happens; and the words “as a means to achieving sustainable development” have become “a contribution towards”, suggesting a toning down of the convention's perceived leadership in that area.

In the run-up to COP XII (2015) the Standing Committee established a working group to prepare a fourth strategic plan for the period of two triennia 2016–2021. Standing Committee requested a shorter, simpler, and more engaging plan, using language and consultation formats, like the other multilateral environmental agreements. However, such calls fell on deaf ears, and the fourth strategic plan, agreed at COP XII (2015), reverted to the error of the second plan by becoming voluminous, with many (unnecessary) pages of introduction. It does have some additional features; importantly running for nine years rather than six originally considered (i.e., 2016 -2024). Specific indicators were identified for each of the targets with the expectation indicators will be monitored by Contracting Parties. Additionally, Standing Committee was to keep the implementation of the strategic plan under review, based on regular reports from the Secretariat, the STRP, and national reports.

### Regional approaches

To provide effective support for improved implementation of the convention, a range of Ramsar Regional Initiatives (RRIs) have been developed since COP 4 (1990), where particular Parties or groups of Parties wished to show leadership, but in a somewhat ad hoc fashion. Regional initiatives allow ready involvement in implementation of the convention of all stakeholders, including all relevant ministries, intergovernmental bodies, IOPs, other NGOs, academia, local communities, and economic actors, while allowing focus on local specificities.

The oldest of the RRIs is the Mediterranean Wetlands Initiative (MedWet), established in 1991, with support from the Barcelona and Bern Conventions, the European Commission, the governments of Italy and Greece, and several NGOs. MedWet brings together 27 Mediterranean and peri-Mediterranean countries that are convention Parties. MedWet’s role is to support the effective conservation of Mediterranean wetlands and the sustainable use of their resources and ecosystem services. Resolution XIII.9 set out the current position for Regional Initiatives—there are currently four regional Ramsar *centres*, focused on training and capacity building and fifteen Ramsar *networks* focused on wetland organisations and governments in regional cooperation. The latter group includes the intergovernmental East Asian-Australasian Flyway Partnership. It is fair to say not all Regional Initiatives are equally effective, and so Resolution XIII.9 decided to re-establish an (open-ended) RRI Working Group to draft new operational guidelines for them and submit their recommendations to Standing Committee and onwards to COP XIV in 2022.

## The dynamics of institutional drifting

### Water seeps into the convention

The second strategic plan suggested it would assist the convention contribute to *inter alia*: the fourth World Water Forum in Mexico 2006 and implementation of decisions from the thirteenth meeting of the Commission on Sustainable Development policies on water and sanitation. These proposed contributions go well-beyond “wetlands and waterbirds” and dip the convention’s toe, figuratively and literally, into water resources. This concretised part of Resolution VI.23 that the convention should “ensure, through partnerships with water related organisations such as the World Water Council, that the Ramsar Convention becomes an audible voice in water debates.” This move, scarcely noticed in 1996, but mainstream by 2005, finally took the convention away from its original objective as a *Convention on Wetlands of International Importance, especially as Waterfowl Habitat* and towards a convention on wetlands and water *sensu lato.*

The preamble of the third plan agreed at COP X mentions specifically: “In the 1960s the driving force behind the establishment of the Ramsar Convention was concern over the continuing destruction of wetlands and the impact of this destruction on populations of waterbirds. Yet, almost 35 years on Millennium Ecosystem Assessment (2005) concluded that “degradation and loss of wetlands (both inland and coastal) is continuing more rapidly than for other ecosystems”. The third plan has many references to water and its management’ including “Cross-sectoral recognition of wetland services and Integrated Water Resources Management” and “Synergies and partnerships with UNECE Water Convention and UN Water”. This emphasis on water issues continues the increasing importance water was playing in the convention discussions. By COP X (2008) it had effectively become a wetlands and water, not a wetlands and waterfowl convention. This emphasis on the centrality of water issues further promoted drift in the convention, with a wetlands and water publication (Russi et al. 2013) within The Economics of Ecosystems and Biodiversity programme (TEEB 2010). Significantly, Russi et al. (2013) was launched at the seventh World Water Forum in the Republic of Korea, not a wetlands or waterfowl focussed event.

Resolution XI.3 added the following terms; water quality, environmental flows, environmental integrity, productive sectors, education to strategy 1.4 of the third plan. Also mentioned in Resolution XI.3 was that “effective management of Ramsar Sites and the wise use of the rest of the world’s wetlands is an essential contribution to the work of the UNECE Water Convention”, as well as emphasising the importance of water-related conventions to Ramsar’s thinking and actions.

A Standing Committee working group developing the fourth strategic plan concluded that “at an overall, global level, implementation of the convention can be characterized as a work in progress”. It noted that several core aspects of the convention, such as the wise use of wetlands, identification of potential Ramsar Sites, inventories, preparation of management plans, monitoring of site status and ecological character, and reporting under the convention “continue to require regular attention and action”—hardly a ringing endorsement after over 40 years of operation. The working group’s other main finding was an increasing sense of urgency amongst Contracting Parties in the face of accelerating degradation and loss of wetlands, and that responding to this requires “mainstreaming wetland values in public and private investments leading to better management of wetlands”. The plan identified 14 priority areas of significance for the convention to follow. Water appears 72 times in this list, waterfowl/waterbirds/migratory species once.

A progress review of the fourth strategic plan will be undertaken at COP XIV in 2022, analysing its progress against *inter alia* the Post-2015 Sustainable Development agenda and Sustainable Development Goals. At COP XIII (2018), Resolution XIII.5 established this formal review process noting that” the review of the fourth strategic plan represents an opportunity to consider the key messages from the Global Wetland Outlook and the approved messages from the Summaries for Policymakers of the IPBES assessments, and to include recommendations in this regard”. This proposed review places much emphasis on further developing CEPA in the convention, as Parties were becoming convinced the “wetland message” was simply not getting out to the wider community and that, as Ramsar neared 50, positive effects from its work were difficult to discern. And yet this is despite significant funding from the private Sector (Danone Company since 1998) for CEPA, especially the annual World Wetlands Day actions and materials.

Although institutional drift is not mentioned as such, in effect that is what will be reviewed. Table 1 shows the extent to which there has been a decline in attention given to site-related matters, and a rise in a wide range of peripheral issues, often disappearing after discussion at only one or two COPs. This shows the extent to which the convention has suffered from drift, aided by strategic plans which add more and more issues, instead of refining and retaining the original focus of the convention. The review process (currently in train) will consider changes needed to craft a fifth strategic plan envisaged from 2024. This is clearly the moment for a complete re-evaluation of the direction of the convention, given its lack of success in achieving its original aims, and the filling of its other possible “niche space” by a range of multilateral environmental agreements and UN programmes. A detailed timetable was established under Resolution XIII.5, for this review, but, as with all global and national processes in the Covid-19 era, this is running well behind schedule.

### The role of the dramatis personae

What the review of the fourth strategic plan is not attempting, but perhaps should, is a review of interactions between the key actors. While the four original IOPs, now with the two later additions, were active in the discussions leading up to the convention’s establishment, and largely working from a strong science base, there has been increasing divergence since 2000 in specialisation and function of the IOPs themselves, their interaction with the convention, and each other.

The IUCN, as an international NGO, has a complicated relationship with the convention. As specified by the convention text, the IUCN supplied the Secretariat functions (with IWRB support) until appointment of the first Secretary General in 1988. At COP4 (1990) the arrangements were formalised for the IUCN alone to act as host for the Secretariat, although the IWRB continued to play a very active role in convention matters. Such hosting should offer “water cooler” possibilities for intense interaction with IUCNs programmes on water and wetlands, ecosystem management, species survival etc. And yet that has failed to develop, despite the very warm words from the IUCN Director General at COP4 (1990) when he noted that the “IUCN attached very great importance to the existence of the Ramsar Bureau (now Secretariat) within the IUCN headquarters and hoped that continued strong partnership between Ramsar and the IUCN would serve to achieve the common goals of the two organizations”. Yet currently the IUCN Global Water Programme on its web microsite has no reference to the convention, rather promoting its own potential for action and knowledge on sustainable water resource management. The IUCN (through its regional Asia Office) does act as the Secretariat for the Indo-Burma Ramsar Regional Initiative (IBRRI), but this is the IUCN’s most direct involvement, apart from the ritual welcome of the Standing Committee to the IUCN headquarters by the Director-General.

During the 1990s—early 2010’s WWF was very active in promoting the addition of new sites under the convention, yet currently no longer has links to the convention on its website, the focus being on freshwater and threatened species. The WWF is also complicated in that in-country branches of the organisation often have quite different emphases from the International Office, the traditional focal point for Ramsar links. Having driven hard for rapid increase in number and size of Ramsar sites it seems as though the WWF is no longer especially interested in the convention, although retaining its IOP status.

Wetlands International has a greater stake in the convention. Until 2015 Wetlands International managed the Ramsar Sites Information Service. There had been increasing unease amongst Contracting Parties concerning this outsourcing, and so, in 2015, the Ramsar Sites Information Service reverted to being hosted by the Secretariat. This newly constructed system enabled Contracting Parties to designate and update Sites online—a considerable improvement. Wetlands International still features the convention on its website, especially noting the importance of the International Waterbird Census (IWC). This census is one of the longest running citizen-science global projects. It covers both threatened and non-threatened waterbird species. Information collected by the IWC has helped the Ramsar Convention STRP develop advice for the Parties in deciding if waterbird populations comply with convention rules for site nomination as an Internationally Important Wetland. This work by Wetlands International also links with activities undertaken by fellow IGO Birdlife International for Ramsar and the Convention on Migratory Species, as well as Birdlife international’s own “Important Bird Areas”.

Given the original importance of waterfowl in convention discussions Birdlife International itself has been involved with the convention since assisting its birth. In recent years however, Birdlife International has moved to a broader canvas. Information on Ramsar on the website is dated (mostly around 2011–2012), and in recent times it has tended to support more the Convention on Migratory Species, and in 2019 and 2021 the successful efforts by China and the Republic of Korea, to have a significant coastal area listed as World Heritage.

As part of the CGIAR system the International Water Management Institute, is different from all other IOPs, and therefore has different approaches to wetland work and values. Its web site has links to a recent blog featuring Ramsar’s approach to gender roles in wetland wise use—a particularly thoughtful and important piece (Joshi 2021). It also reacted well to the Wetland City accreditation process, established in 2015 in Resolution XII.10. Such enthusiasm is unsurprising since its seat is Sri Lanka and Colombo was one of the first “wetland cities” accredited under the convention. The role of IWMI in promoting these issues is an important part of the convention’s evolution.

As the CEO of the Wildfowl & Wetlands Trust (WWT) noted on its acceptance as an IOP in the report of COP XIII (2018) “it was surprising that the organisation had not previously been so accepted”. Based in the UK, WWT has become more and more international in focus. In working with Ramsar, WWT has brought two important initiatives. The first relates to the developing CEPA programme of the convention—especially through local wetland centres at Ramsar sites. This is the Wetland Link International initiative (WLI) that aims to improve the role of its over 350-member wetland centres in changing attitudes, promoting good wetland conservation, and educating and informing local stakeholders and visitors. A second initiative is the World Wetland Network (WWN) that aims to engage and support local NGOs during COPs, with a Pre-COP meeting, organising, and promoting statements of civil society groups, and working with Parties and NGOs on draft COP resolutions. This initiative has been valuable in uniting the vast number of local wetland NGOs.

As can be seen from the foregoing, the current linkage between IOPs and the convention activities, and between the IOPs themselves, have changed markedly from the early years of the convention. While IOPs have had an important role in shaping the evolution (and success or otherwise) of the convention, there are many external bodies that also play key roles in shaping convention work. The convention website currently lists 25 Organisations with which an exchange of signed letters, a formal Memorandum of Understanding (MOU), Memorandum of Cooperation (MOC), or a Joint Work Plan (JWP) is in force. Yet the effectiveness of all 25 is open to question, and in some cases is plainly window-dressing. Important areas where formal cooperation occurs frequently are with the Convention on Biological Diversity through a Joint Work Plan; the Liaison Group of the Biodiversity-related Conventions; and the Convention on the Conservation of Migratory Species of Wild Animals. Membership of the UN-Water group by the convention is as a “partner”, alongside many of the convention’s own IOPs. This status is exacerbated by not being a UN convention.

### Absent actors

Despite this impressive set of actors, there are important actors missing. Notably, Indigenous Peoples and Local Communities (IPLCs). The Convention on Biological Diversity, and the Intergovernmental Platform on Biodiversity and Ecosystem Services have had tense, but increasingly profitable, interactions with IPLCs. Yet the convention has not fully realised its potential in this area, despite a long history of attempting to create better relationships with IPLCs.

In 1996 at COP 6, Recommendation 6.3 called upon Contracting Parties “to make specific efforts to encourage active and informed participation of local and indigenous people, at Ramsar listed sites and other wetlands and their catchments, and their direct involvement, through appropriate mechanisms, in wetland management”. A particularly poignant response to this came at COP VII (1999) in Costa Rica, where during the meeting representatives of IPLCs in Mesoamerica laboured daily on an enormous painting showing the values of wetlands in the region, artistically based upon the fishing culture of the Solentiname archipelago in the southeast of Lago de Nicaragua. At the conclusion of the COP, the artists presented their painting and their "People's Declaration on Wetlands" during the closing ceremonies. The Ramsar website (Ramsar 1999) notes that “In the rush of events this (the declaration) was not included in reports of the COP and never made available on this Web site. The Declaration *inter alia* noted that “ Parties should:

- Create and apply all programs in accordance with the reality experienced by each one of our (IPLC) communities;
- Increase awareness in populations and provide training and incentives for the sustainable use of wetlands resources;
- Ensure the prompt and just application of legal frameworks in our countries;
- Promote community self-help in order to seek problem-solving choices based on their own reality.”

Attempts made at COPs VIII (2002) and COP IX (2005) to deal with IPLC links to wetlands through the broader issue of “culture” did not achieve major changes to IPLC recognition in the convention. An informal Ramsar Culture Network, including but not limited to IPLC involvement, has existed for several years but failed to make progress. Involvement of IPLCs thus lay largely unresolved until COP XIII (2018), where Resolution XIII.15 built on earlier resolutions and emphasized “that environmental, social and cultural solutions including those of indigenous peoples and local communities will all be needed to achieve climate change targets”. While this does indeed note IPLC interaction with wetlands, it is done through the prism of climate change, and effectively continues the marginalisation of IPLC roles in wetland management.

A report for the convention (Oviedo and Ali 2018) should help focus attention and it is to be hoped COP XIV (2022) will tackle this issue more effectively and proactively. Debates provoked by suggestions that wetlands should be accorded rights (Davies et al. 2020; Bridgewater 2021) also play into this discussion. Certainly, in this fiftieth year of the convention it is time to accord better recognition of the role IPLCs have had, and continue to have, in wetland management and conservation in the 25% of the earth’s surface under their direct control (IPBES 2019).

### Wetlands and Sustainable use

At Standing Committee 42 (2011) the CEO of Wetlands International outlined a daunting array of challenges to people’s livelihoods around the world and described wetlands as being on the “front line” of the key development and security challenges that the world faced. She described several ways in which the IOPs can add value in all this work by bringing their experience from programmes and supporting effective policy development. This showed the influence IOPs were hoping to exert in the third strategic plan, and the extent to which IOPs were moving from science-based nature conservation organisations to ones advocating for sustainable development and poverty reduction.

Several text additions were made to the third strategic plan at COP XI (2012), viz. “achievement of Millennium Development Goals; achievement of the Aichi Biodiversity Targets of the Strategic Plan for Biodiversity 2011–2020” (CBD COP X Decision X/2). Interestingly the convention mission was also elaborated by the addition of the following codicil: “To achieve this Mission it is essential that the vital ecosystem services, and especially those related to water and those that wetlands provide to people and nature through their natural infrastructure, are fully recognized, maintained, restored and wisely used.” It can be argued that the IOP influence on the Parties’ direction of travel was now clear, and that the transformation of the convention from waterfowl convention to water convention is now complete. This strategic plan was agreed as the world accepted the Sustainable Development Goals and the 2030 Agenda.

## Conclusions

**Fig. 2:**
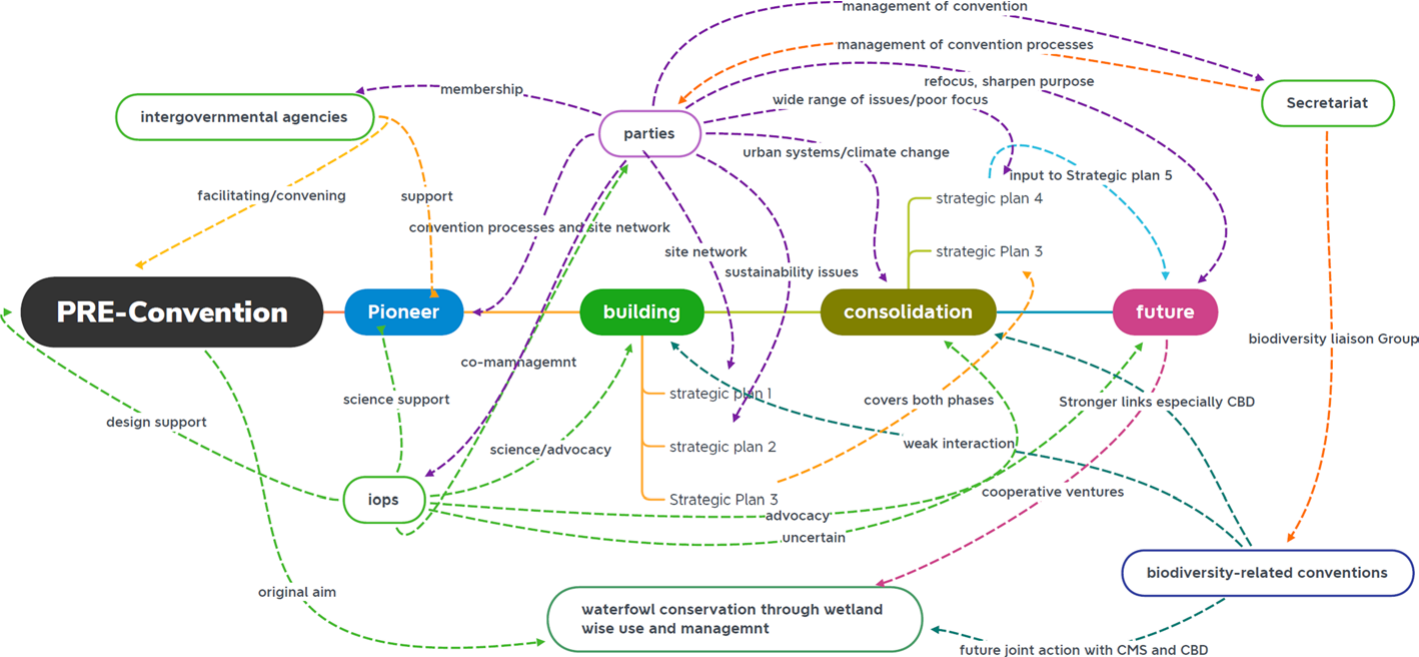
A conceptual framing of the processes and actors that have influenced the convention’s development from its formation phase, through the three periods of convention activity and projecting to the future. An element omitted is that of interaction between Secretariat and International Organisation Partners. Such interactions do take place informally, as well as more formally through Contracting Parties

Our historical account of the evolution of the Ramsar Convention offers a basis on which a new theoretical framework for treaty evolution could be developed. While the literature mostly puts emphasis on how treaties evolve through the will (or lack thereof) of the contracting parties (Sand 1997; Gehring 2007; Bodansky and Diringer 2010; Brunnée 2012; Bodansky and Rajamani 2013), our study has found that treaty evolution is a much more multifaceted process than one would expect. We find that treaty evolution is heavily influenced by both internal and external actors with which central treaty bodies interact (Biermann and Siebenhüner 2009; Jinnah 2014), or more broadly by the institutional environment in which a treaty operates (Biermann and Kim 2020; Abbott et al. 2016; Kim and Morin 2021; Kim 2020). In explaining treaty evolution, it is then important to look beyond the politics of member states, and to the constellation of actors and interests in and around the convention. As the foregoing text suggests, for the Ramsar Convention this is a complex picture. Figure 2 attempts to illustrate how these different components of the convention have contributed to its current state.

What is important to note is that these internal and external actors themselves evolve, which in turn affect the evolutionary dynamics of a treaty. We suggest that such co-evolutionary dynamics may explain institutional drifting (or maladaptation) of a treaty, giving an appearance of success yet drifting to failure. In the case of the Ramsar Convention, two key conditions for institutional drifting we identified are: (1) its unique situation as a non-UN convention, which served as a significant blockage to the convention playing a fully complete and competitive global role; and (2) the increasing number of conventions dealing with biodiversity issues, which forced the Ramsar Convention to seek different roles in an increasingly competitive institutional landscape.

To remedy this drifting, Bridgewater and Kim (2021) alluded to the need to use the UN Sustainable Development Goals to realign convention thinking. Emphasising a broader wetscape approach, wetlands in the broader landscape matrix is another profitable area for the convention into the future (see also Tickner et al. 2020), as well as “blue carbon” as the key ecosystems involved are covered under the Convention. This suggests a profitable link to the UNFCCC where the role of wetlands needs highlighting to balance the hegemony of forests as the nature-based solution to mitigate climate change. As nature-based solutions, through conservation and wise use wetlands and their contributions to people (Pascual et al. 2017) offer both mitigation and adaptation pathways to achieving the Paris Agreement (Thorslund et al. 2017).

Embracing these ideas, but with a strong focus on returning to the convention origins—the need for better global conservation of feeding and breeding spaces for waterbird species, may be the most viable path for the next 50 years. The third strategic plan specifically mentions waterbird flyways and encourages Parties “with shared basins and coastal systems to consider participation in regional site networks and initiatives in place for additional wetland-dependent migratory species, as exemplified *inter alia* by the African-Eurasian Migratory Waterbird Agreement (AEWA), the East Asian-Australasian Flyway Partnership (EAAFP), the Western Hemisphere Shorebird Reserve Network, and the Central Asian Flyway Initiative”. Yet these flyway agreements have become effectively outside the purview of the convention. The convention’s role in conservation of wetlands and waterfowl, however, can provide for the physical spaces for the birds in a way those agreements cannot. Future cooperative joint arrangements between flyway agreements, the Convention on Migratory Species, the Convention on Biological Diversity and Ramsar would seem to have potential for solving many of these issues. While all conventions suffer from drift and mission creep, in the case of the Ramsar Convention we assert the most viable future lies in returning to its origins, while engaging in novel issues of the twenty-first century where they can improve the lot of migratory waterfowl. For example, combining a focus on blue carbon and the conservation of coastal site for breeding and feeding is an obvious direction of travel.

The third plan also, for the first time, incorporated a vision: “Wetlands are conserved, wisely used, restored and their benefits are recognized and valued by all”. Such a vision, embracing the aims of the convention as agreed in 1971 and rewritten in more usual vision style might be “A world in which wetlands are conserved and wisely used for the benefit of people, waterfowl, and the rest of nature”. With such a vision, the imperative for the next decade would be to arrest institutional drift, return to the original purposes of the convention in the context of twenty-first century knowledge, and succeed in joining the UN system. In that way, returning to the convention’s founding ideals in a stronger fashion can only benefit people, birds, and the rest of biodiversity in a much more effective way.

## Data Availability

All material used is available through websites.

## Footnotes

1 The differing percentages in these assessments relate to the period under consideration—but both present the same dramatic decline of wetland area, accompanied by decreasing quality of much of the remaining wetlands.

2 The number of the COPs and their recommendations and resolutions follows that of the convention, i.e., Arabic numerals until the sixth, after which Roman.
